# Antibodies against West Nile and Shuni Viruses in Veterinarians, South Africa

**DOI:** 10.3201/eid2008.131724

**Published:** 2014-08

**Authors:** Charmaine van Eeden, Robert Swanepoel, Marietjie Venter

**Affiliations:** University of Pretoria, Department of Medical Virology

**Keywords:** West Nile virus, Shuni virus, veterinarians, South Africa, zoonoses, viruses, arboviruses, vector-borne infections

**To the Editor**: Many arboviruses are zoonotic; humans acquire infection from the bites of arthropod vectors or through exposure to the tissues and body fluids of infected animals. West Nile virus (WNV), a widely endemic zoonotic agent in South Africa, occurs wherever the principal vector (*Culex univittatus* mosquitoes) and avian hosts are present (*1*). Serosurveys based on hemagglutination inhibition and neutralization assays conducted during 1950–1970 indicated that 17%–20% of long-term rural residents in the Karoo, 4%–8% in the Highveld, and 1%–3% in the Natal and the Eastern Cape areas had antibodies against WNV (*1*). Most human infections tend to be sporadic and are characterized by mild febrile illness (*2*); however, severe disease has been documented (*3*). WNV has caused severe neurologic disease of horses in South Africa (*4*), and zoonotic transmission was recorded in a veterinary student who performed a necropsy on an infected horse (*5*). 

Shuni virus (SHUV) (genus O*rthobunyavirus*, family *Bunyaviridae*) was first isolated in Nigeria in 1966 during surveys of livestock, *Culicoides* midges, and mosquitoes, SHUV also once was isolated from a febrile child (*6*,*7*). SHUV recently was identified as a previously undetected cause of neurologic disease in horses in southern Africa (*8*) and is thus of interest in comparison to WNV.

To determine the potential for human infections, we tested veterinarians as a high-risk group for evidence of infection with these 2 viruses. Veterinarians with regular exposure to horses, livestock, or wildlife—and thus to vectors because of an outdoor lifestyle—were invited to donate blood samples at specialist veterinary conferences in South Africa in 2011 and 2012.

The Kunjin MRM61C strain of WNV (*9*) and SHUV isolate SAE 18/09 (*8*) were cultured and harvested when the cytopathic effect (CPE) reached 80%. Stock virus was titrated in 100-μL volumes in 6 replicate wells per serial 10-fold dilution (10^−1^ to 10^−9^) in Leibowitz medium with 5% fetal calf serum (Invitrogen, Carlsbad, CA, USA), with 100 μL of medium added in place of test serum dilution, and 25 μL of Vero cells (8 × 10^5^ cells/mL) added per well. Plates were incubated at 37°C; CPE was monitored; and 50% tissue culture infectious dose per milliliter endpoints were calculated. For neutralization tests, serum were inactivated at 56°C for 30 min; duplicate 100-μL volumes of doubling dilutions were prepared in Leibowitz medium (1:10–1:640) and incubated with equal volumes of medium containing a calculated 100 50% tissue culture infectious dose virus at 37°C for 45 min, and 25 μL Vero cells (8 × 10^5^ cells/mL) were added per well. Medium was added in place of virus to replicate test serum controls at a 1:10 dilution to monitor for toxicity of the serum, and the virus used in the test was back titrated. Tests were monitored for 10 days. Neutralization titers were expressed as the reciprocal of the serum dilution that inhibited >75% of CPE in both replicates, and only titers >20 were recorded as positive for virus antibodies.

Serum samples were received from 123 veterinarians in South Africa and 4 from neighboring countries. Ten (7.9%) serum samples tested positive for antibody to WNV and 5 (3.9%) for antibody to SHUV; all positive serum samples (titers 20–80) were from South African veterinarians. Prevalence of WNV antibody in men (5/81 [6.2%]) and women (5/46 [10.9%]) did not differ significantly. The veterinarians ranged in age from 23 to 71 years and had practiced an average of ≈23 years; the prevalence of WNV antibody was similar in age groups23–50 years (6/74 [8.1%]) and 51–71 years (4/53 [7.5%)].

Most veterinarians came from periurban practices in Gauteng (51/123) and Western Cape Provinces (18/123); the comparatively small numbers of samples from elsewhere preclude valid comparisons with the historical surveys of rural residents. However, indications that veterinarians might be at increased risk for infection in some areas included a 23.1% (3/13) prevalence of WNV antibody in KwaZulu-Natal veterinarians and a similar prevalence of antibody in much smaller sample groups in the Free State and Northern Cape Provinces. In Gauteng, where most horses reside, 6% of veterinarians tested positive for WNV, which reflects the prevalence described for the Highveld region in the 1970s.

Four of the 5 veterinarians positive for SHUV antibody were men; 2 were in the 23–50-year age group, and 3 were in the 50–71-year age group. Of the veterinarians who tested positive, 3 were identified in Gauteng (3/51 [5.9%]) and 1 each in the Eastern Cape (1/8) and Limpopo (1/8) Provinces. No clear histories of disease compatible with the infections could be elicited from any of the veterinarians whose samples contained antibodies. Nevertheless, the 2 viruses, and related arboviruses, tended to be overlooked as animal and human pathogens in southern Africa until recently, and greater awareness is needed of their potential as zoonotic agents. Investigations of neurologic illness in humans identified several WNV cases that had been overlooked in hospitals in Gauteng (*10*). Similar investigations of febrile and neurologic illness in humans might shed light on the possible clinical significance of SHUV infection in humans.
